# Veterinarians as Sanitarians1Read at the meeting of the Tennessee Veterinary Medical Association, held at Chattanooga, Monday, October 24, 1898.

**Published:** 1899-03

**Authors:** G. B. Blackman

**Affiliations:** Blackman, Tenn.


### VETERINARIANS AS SANITARIANS.[1]

By G. B. Blackman, D.V.S.,
BLACKMAN, TENN.

Time and experience test the work of men, and the highway of progress is covered with fragments of countless inventions. The creeds, the dogmas, and social relations of one age become the by-words or the antique curiosities of the next. Men do what they can, and coming generations pardon their errors, bnt judge their work as they ought. What is good lives; what is bad dies. This is the general rule. We are living in an age that demands of every man the best fruits of his mental and physical energies to meet the exigencies of the times. The closing years of the nineteenth century are the ripening stage of the greatest minds that have ever lived. As the months roll by, responsibilities multiply upon the shoulders of those who think and work hardest in the ranks of the professional, architectural, and scientific classes. The struggle is to reach the high peak of human endeavor. Never before have such auspicious opportunities for usefulness and preferment among its members been laid upon the threshold of our profession as are there to-day. The man of ability and thoughtfulness readily appreciates the situation, and with willing hands will grasp the rough material, fresh from the sources of inexhaustible supply, in the professional field, to mould and to polish the roughened stone into a character of usefulness, beauty, and perpetuity. A profession that within a short time has been looked upon with an air of disdain and ridicule by men of other professions is now beginning to be recognized and accepted as a factor beneficent in the great problem of social economy. Civilization is gaining friends hourly, and enlarging its borders daily. It is utilizing forces that hitherto were latent; that which in the past was refused is now material of usefulness and adornment.

As individual members of the veterinary profession we find ourselves in the midst of momentous duties—responsibilities which are not altogether new, but are forced to the front, demanding a solution as one of the problems incident in our Commonwealth. The question of sanitary science and its practical application by the profession is confronting us with irresistible force.

In no small degree are the health and wealth of the nation depend-

[1] Read at the meeting of the Tennessee Veterinary Medical Association, held at Chattanooga, Monday, October 24, 1898.

ent upon our ability and energy; true, it is not appreciated in all the States as yet, for in some of them there are no laws governing the suppression or eradication of infectious diseases. Are we equal to the emergency? The result of our professional career will best answer this question. The consumers of meat and dairy products are largely at the mercy of tradesmen, with a few exceptions, of firms whose sense of honor and justice in their business relations with the public is based on the broad principle of dealing with others as they would be dealt with. It is this class of business men and the veterinarians that the millions in our States who daily consume meats and dairy products can only depend upon as the proper persons to protect their tables from unwholesome food. The competition in the manufacture and sale of food-products is exceedingly spirited these days, and this rivalry does not always tend to increase the merit and quality of the articles. Then the spread of diseases among animals themselves, and the many avenues by which diseases are transmitted from the lower animal to mankind, their detection, prevention, and eradication are questions, all of which require a solution at our hands.

There is no one article of food so universally used as that of milk. It possesses all the elements necessary for primary growth; it is the initial and only food for the newly born, and continues as such for months. One of its physical characters is its susceptibility to untoward influences. It will receive and convey the odors of its surroundings. The unpleasant flavor of certain vegetables, or sour fermentative foods which the cow may eat, are readily detected in milk, and of all the foods that reach our table none furnish a more genial habitation for nearly all forms of bacteria than milk.

Not a year passes that we do not have living proof of the communicability of disease from various sources through the medium of milk, diseases that are not wholly infantile, but attack man at any age—typhoid fever, tuberculosis, common measles, German measles, and others. Not only has it been proved that certain diseases incident to the bovine family have been carried through milk to man, but that diseases wholly allied to man have been transmitted through this same medium; thus this article is open to a double source of infectiousness, and of the two sources of infection, we find that in man lies the greater evil of the two.

Aside from bovine phthisis, there are but few diseases of frequency in that family considered dangerous and liable to transmission through dairy products; but in man we have a number of infectious diseases which can be transmitted through this medium.

Whether tuberculosis in man has its essential source in the bovine family or not, there are, however, certain conditions of the disease in the animal which seem even on the surface to be inimical to health, and when considered from the ground of its possible relation and potency toward producing the disease in man there is at once, of itself, a wall of defence very difficult to destroy. We have intimated that by men milk is environed with contaminations which lay it open to serious and fatal consequences ere it reaches the consumer; the wonder is it is not more so. A daily food so well suited for the abiding place of germ-life, which develops and multiplies indefinitely, requires more than ordinary attention while in the hands of the producer and tradesman.

There are many cases on record which are undeniable proof of the spread of typhoid fever, diphtheria, and many forms of enteric diseases through milk alone. One prominent case in proof of the foregoing statement: In Stamford, Conn., in April, 1896, typhoid fever made its appearance in the early part of the month. No special significance was attached to its invasion at first, as at that time but few cases had developed, but before the month was gone 209 cases had been reported to the board of health. Diligent search was made to find the cause of such havoc, and before the fell destroyer was found, and from whence it came, it had as its claim 386 victims and 22 deaths. A paragraph from one of the New York papers said: "Every one of these cases has been traced directly to a foul well in the stable-shed of H. R. Blackham, a retail milk-dealer, on Greenwich Avenue in that part of the town known as 'Water Side.' A map was made showing the location of the cases and Blackham's milk route. The doctors have not driven off of Blackham's route in their visits. Every house with typhoid patients was a stopping-place for Blackham's horse. Dr. T. Mitchell Prudden analyzed the water from Blackham's well, and says, 'The number of living bacteria of various kinds in 1 c.c. of this water is 69,660. This number of germs would be reasonable in sewer-water or a cesspool.'" How often it is the case that the family physician can trace the enteric trouble of the babe to the milk-can; and not infrequently do we hear of tubercular meningitis in the child of a family whose record is free from the disease, and upon investigation the source is traced to the family cow or the dairy from which they had long purchased milk.

It is along this line that the veterinary surgeon can make for himself a reputation worthy of his calling. In no other way can he manifest greater skill than in being able to remove the cause of disease, rather than treating it successfully; and especially will this prove true at the dairy-farm, the products of which are placed on the market. There is a great need of an entire reform in the management of abattoirs and dairies in our State. And who, among the scientists, is better qualified to perform so important a public function than the veterinarian?

I will not go into the details of the dairy, which are many and complex. Aside from dairy-products, we will mention meat-markets or the sale of meats. What should be done with an actinomycotic animal? Learned men in the profession differ as to the contagious character of this disease, except among bovines, and I believe this is disputed by some. However diversified opinion may be regarding this disease, is it not well to give the doubt the preference? I would not be understood as sentimental, but I believe there are occasions when intuitive knowledge or science, or, more plainly put, instinct, is to be relied upon, when doubt and ignorance abound. What man possessing a cultured sensibility, to say nothing of professional knowledge in the medical science, can make himself believe that the flesh of a diseased animal, though it be only localized, receiving as it does its blood-supply from the systemic circulation, giving off its effete material in the form of a ptomaïn, to be partially eliminated through the process of arterialization in the lungs, and then the remaining fragments sent again to all parts of the body, availing themselves of every opportunity for lodgement in the tissues, though they be attenuated in vital form, must not in time make general the same disease that to outward appearance seems only local? The question of economy should be subordinated to that of health. We cannot admit that cooking is a sufficient guarantee that meat is properly sterilized. It has been shown by investigation that in many diseases it is not the characteristic bacilli of the disease which produces the toxic infection manifesting the symptoms of the disease, but that it is a sub-product, the dead bodies of the bacteria, the ptomaïn, a soluble alkaloid readily absorbed, capable of producing septic intoxication. Nearly all authorities agree that bacilli present in the blood of tetanic patients are few; and in animals in which this disease was produced artificially the blood was often found sterile, the microbes being found at the seat of primary infection, and in the tissues between it and the spinal cord rather than in the blood itself. We must, then, conclude that the laboratory from which these ptomaïns come is in the tissues in which the localization takes place, and it differs not whether the disease is local or general if of importance and contagious in its nature; the blood and tissues possess the septic intoxication which is carried by the presence of dead tissues in the body in a state of putrefaction from the presence of putrefactive bacilli, and that the immediate cause of the intoxication is the absorption of pre formed ptomaïns from such a local focus of putrefaction.

Are we justified in the light of present knowledge, touching so vital a question, by silence to assent to the sale of food-products contrary to revealed knowledge? Or if, on the other hand, our busy life hinders personal investigation, that we may know from experience these facts, are we to doubt the result of the investigation made by others who have investigated?

When human life and happiness hinge on any one question, too much is at stake to admit of theory, long drawn-out deduction, and hair-splitting conclusions to prove that under such and such conditions certain agencies and elements are harmless. Nothing less is expected of us. We are in position to lead and become the sanitarians of our time. Surgical sanitation means more than ordinary cleanliness. It means perfect sterilization of hands and instruments and in a diversified practice this is more imminent as it brings in contact with the medium a greater number of avenues through which to spread infection. The need in the profession to-day is a closer application of sanitary principles to the treatment as well as the prevention of disease—preventive measures based upon pathology.

The influence of inheritance, the transmission of functional peculiarity associated with this inheritance, and the transmission from parent to offspring of certain conditions apparently pertaining to the tissues and predisposition to disease, is a question for concern as vital as treatment of disease, when precipitated.

We can no longer cast aside the necessity of the hour. We are in the field of activity; we must perform. It becomes us, then, to do our part well, take on the harness of rich opportunity, toil for the benefit of our race, and at the end of labor reap reward.

---

Malarial Plasmodium. According to the *Microscopical Bulletin*, the blood of American soldiers in Cuba and Porto Rico, suffering from pernicious malarial fever, was, upon microscopical examination, found to contain the plasmodium malariæ in a crescentic form, thus differing quite radically from the form this plasmodium assumes in temperate latitudes.

